# Bronchomalacia Complication Following a Bentall Procedure in a Patient With Marfan Syndrome

**DOI:** 10.7759/cureus.100072

**Published:** 2025-12-25

**Authors:** Theodoros Milas, Nikolaos Koumallos, Evangelia Sigala, Antonella Koutela, Nikolaos G Baikoussis

**Affiliations:** 1 Cardiac Surgery Department, Hippokration General Hospital, Athens, GRC; 2 Cardiac Surgery Department, El Greco Medical Centre, Lefkosia, CYP; 3 Nursing Education Department, Evangelismos General Hospital, Athens, GRC; 4 Cardiac Surgery Department, Ippokrateio General Hospital of Athens, Athens, GRC

**Keywords:** aortic valve regurgitation, bronchomalacia, cardiac surgery, marfan syndrome, post-surgical complication

## Abstract

Handling extensive aortic aneurysms poses a complex challenge in the realm of clinical practice. Nonetheless, this case study demonstrates the successful surgical management of an aortic aneurysm in a 35-year-old patient with an undiagnosed syndrome related to connective tissue disorder. Remarkably, as a postoperative complication, the patient developed bronchomalacia, which was diligently managed throughout the treatment process. The approach to this distinctive case entailed meticulous surgical planning and skillful intervention, effectively addressing both conditions. Importantly, the successful outcome underscores the significance of a comprehensive and multidisciplinary approach in achieving favorable results for intricate cases of this nature.

## Introduction

Bronchomalacia is a rare and underrecognized cause of respiratory compromise, characterized by dynamic collapse of the bronchial lumen secondary to structural airway weakness [1]. Although mild reductions in airway caliber are frequently seen incidentally, clinically relevant severe airway collapse (>90% reduction in cross-sectional area) is rare and often overlooked unless dynamic imaging or bronchoscopy is performed [2]. Significant symptoms include dyspnea, cough, inability to clear secretions, recurrent respiratory infections, and even respiratory insufficiency. However, formal epidemiological data on incidence in the general adult population are limited, and reported prevalence varies widely depending on diagnostic criteria and patient selection [3].

Although bronchomalacia can develop (or be unmasked) following mechanical ventilation (MV), it is regarded as an acquired and rare complication, supported by indirect but acknowledged evidence [4]. If bronchomalacia develops in an intensive care unit (ICU), it can result in refractory hypoxemia, lobar or total lung atelectasis, trouble weaning off of MV, and extended stays in the ICU [4]. These outcomes are comparable in severity to other known postoperative complications like prolonged ventilator dependence, pneumonia, and acute respiratory distress syndrome (ARDS) [2].

The Bentall procedure is a well-established operation for aortic root pathology, with recognized complications including bleeding, coronary ostial complications, stroke, and arrhythmias [5]. Pulmonary complications are usually attributed to atelectasis, pneumonia, or diaphragmatic dysfunction, while airway structural abnormalities are not considered typical sequelae of the procedure [6]. Nevertheless, several mechanisms may plausibly link major aortic root surgery to the development of bronchomalacia. These include extensive mediastinal dissection in close anatomical proximity to the tracheobronchial tree, prolonged endotracheal intubation, postoperative MV, and altered airway mechanics [1,4]. To date, evidence linking Bentall surgery specifically to bronchomalacia remains limited to isolated reports, underscoring both the rarity of this complication and the importance of detailed case documentation [6-8].

Herein, we present a case of a patient successfully managed with non-invasive mechanical ventilation (NIV) to address this complication.

## Case presentation

A 35-year-old female with a history of mild intellectual disability (ID), kyphoscoliosis, arterial hypertension, thinning of the ventricular septum (potentially linked to a closed ventricular septal defect), and an aneurysm of the left subclavian artery presented with aortic root dilatation (ARD) and aortic valve regurgitation (AR). There were suspicions of an undiagnosed connective tissue disorder (undiagnosed Marfan syndrome) [9]. A successful Bentall procedure was performed at our hospital, involving the replacement of the ascending aorta and root with a 25 mm mechanical valve graft [10].

**Figure 1:**
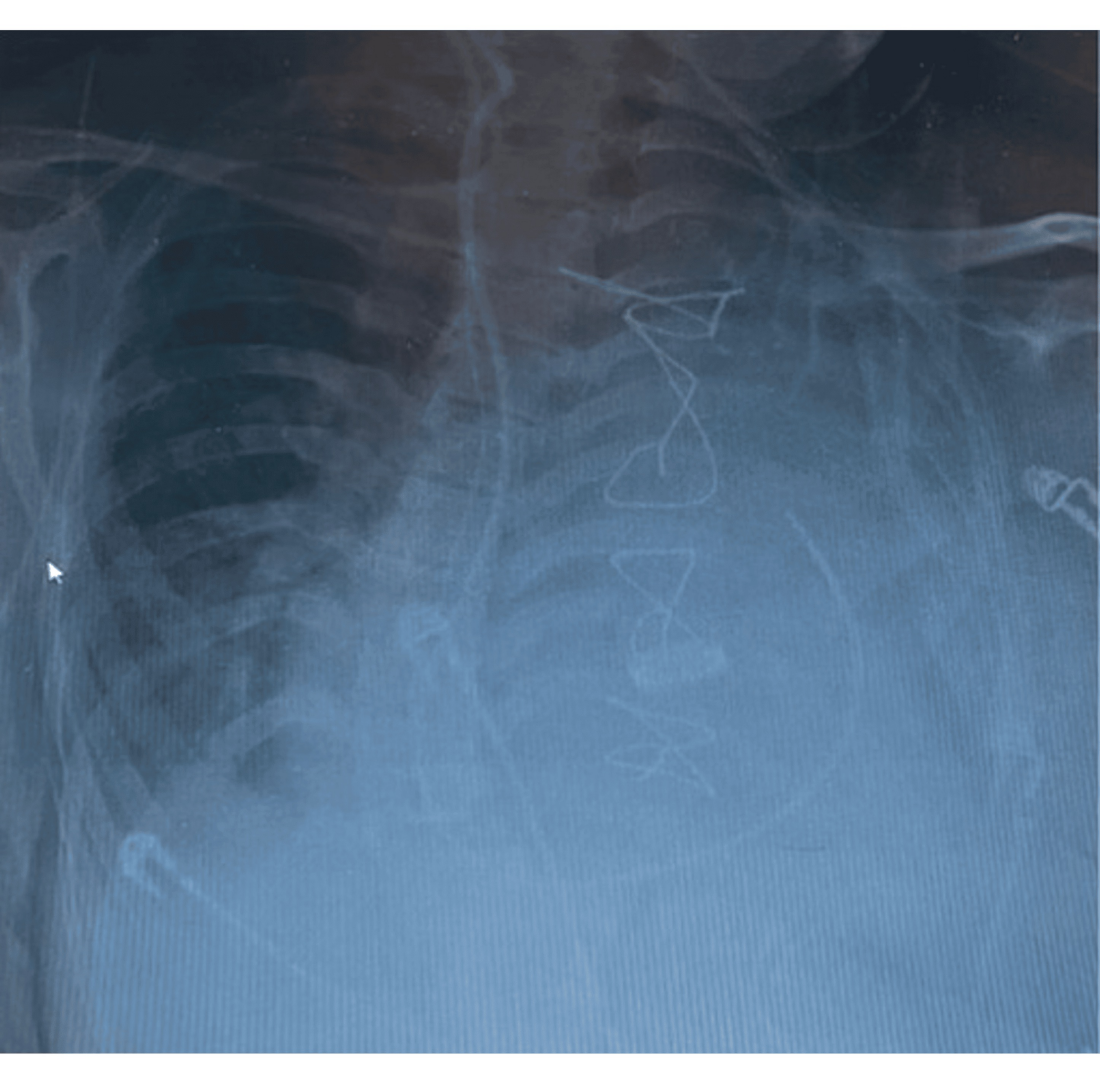
Postoperative chest X-ray showing respiratory failure. The image shows bilateral patchy air-space opacities predominantly involving the lower lung zones, more pronounced on the right side, with reduced lung volumes, consistent with postoperative pulmonary complications contributing to impaired gas exchange.

However, 24 hours after extubation, the patient experienced a collapse due to respiratory failure and tachyarrhythmia, necessitating reintubation (Figure 1). At the time of clinical deterioration, vital signs revealed oxygen saturation (~85% on supplemental oxygen), respiratory rate (>30 breaths/min), supraventricular tachyarrhythmia (heart rate of ~ 140 beats/min), and hypotension (blood pressure ~75/40 mmHg). Arterial blood gas analysis demonstrated acute hypoxemic respiratory failure, prompting urgent reintubation.

**Figure 2:**
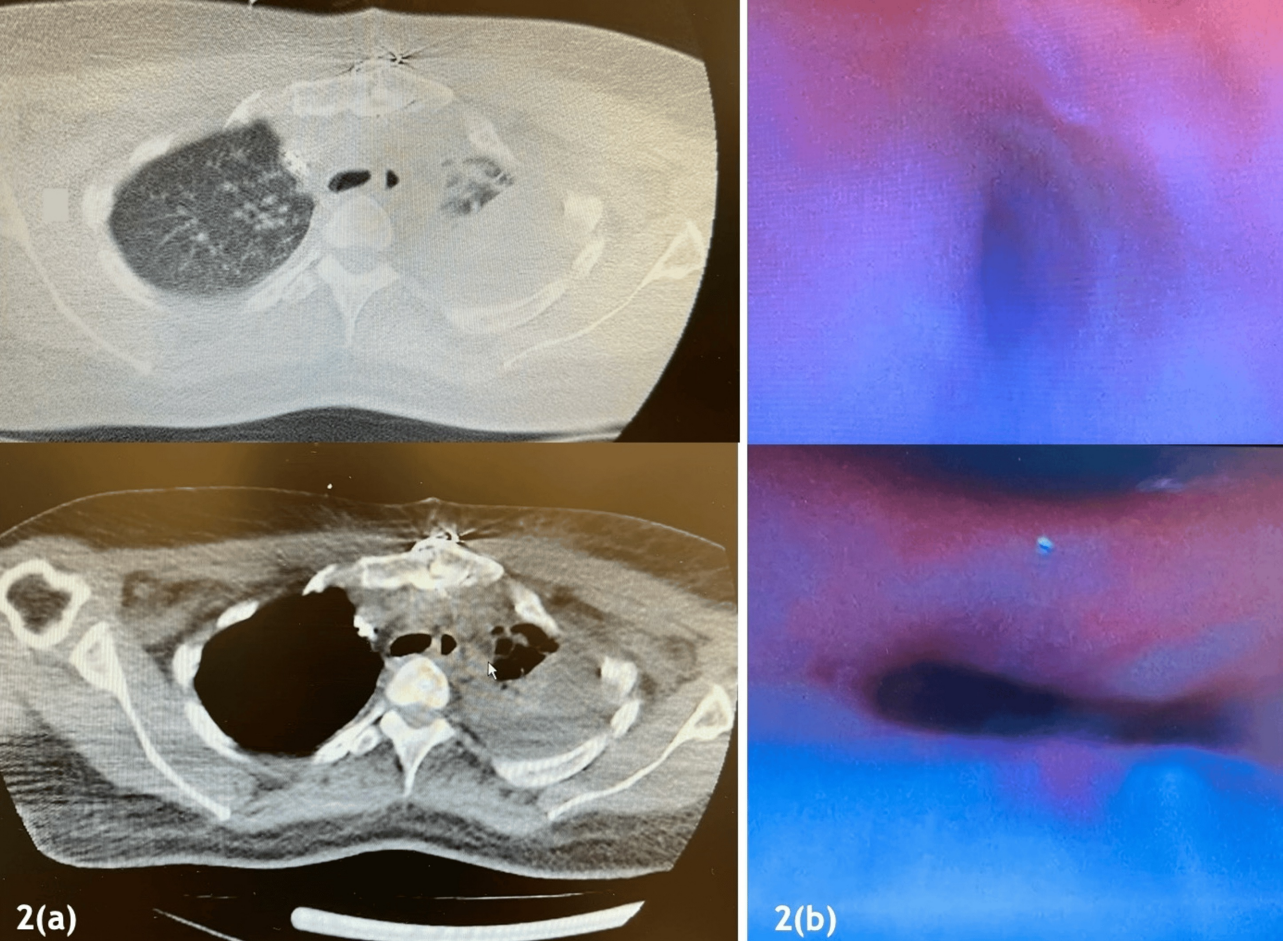
CT (a) and bronchoscopy (b) revealing total collapse of the left lung. (a) Axial chest CT images demonstrate complete collapse of the left lung (arrow), with near-total opacification and marked volume loss, and compensatory hyperinflation of the right lung. (b) Bronchoscopic images reveal complete obstruction of the left main bronchus.

Subsequently, the patient was recommended for a bronchoscopy to address the bronchial compression using a stent device (Figure 2b). During the procedure, a diffuse collapse of the bronchial walls was observed, involving the left main bronchus and extending to the segmental bronchi, which was inconsistent with focal extrinsic compression. This finding led to the abandonment of stent placement and raised strong suspicion of bronchomalacia, a condition previously associated with Marfan syndrome in the literature [7,8]. Initially, the patient received MV followed by NIV for treatment. The weaning from ventilatory support was difficult with recurrent desaturation episodes during spontaneous breathing trials, further supporting the diagnosis of dynamic airway collapse.

A follow-up bronchoscopy was performed, and the final decision was made against stent placement due to severe airway stenosis. As a result, NIV was continued, and alongside this, the patient underwent rigorous respiratory physiotherapy and received close clinical monitoring. Over the next days, the patient demonstrated gradual improvement in oxygenation and tolerance of spontaneous breathing.

**Figure 3:**
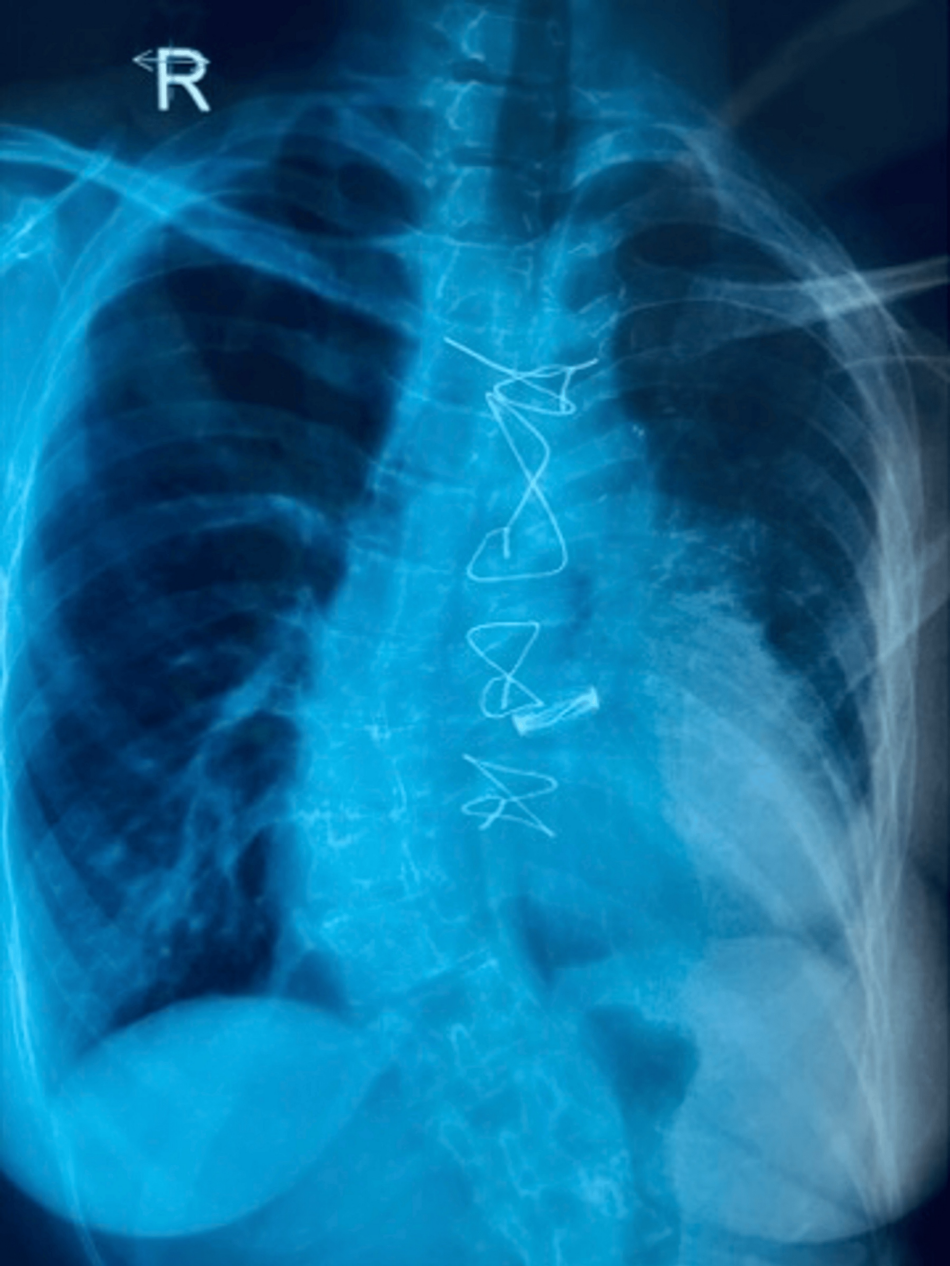
Chest X-ray at four months post-surgery. The radiograph shows re-expansion of the left lung with restoration of lung volumes and resolution of previously noted air-space opacities. There is no evidence of recurrent lung collapse or new pulmonary infiltrates, indicating satisfactory postoperative recovery.

After 35 days of hospitalization, the patient was discharged in a fully mobilized state, with instructions to continue NIV treatment. Notably, six months post-surgery, the patient has not required rehospitalization, and her clinical condition remains satisfactory (Figure 3).

The Heart Team directed the patient and their family to a specialized center for expert genetic evaluation and management [10].

## Discussion

We presented a case of a patient with undiagnosed Marfan syndrome who underwent a successful Bentall surgery but developed bronchomalacia postoperatively. Our suspicion of undiagnosed Marfan syndrome was based on several factors [11]. Notably, the patient displayed distinctive physical characteristics consistent with the syndrome, including a tall and thin stature, various facial features, kyphoscoliosis, long arms and fingers, long legs, and flat feet. Additionally, her relatively young age (35 years old) and the presence of cardiovascular abnormalities (ARD, AR) further supported our suspicion. However, the patient tested negative for FBN1 mutations. Furthermore, family screening did not indicate any genetic abnormalities [9]. Despite negative genetic testing, this presentation is consistent with a suspected syndromic heritable thoracic aortic disease, in accordance with current ESC guidelines [5], with Marfan-like features [9].

The term "bronchomalacia" refers to the weakness and easy collapsibility of one or both mainstem bronchi, without involvement of the trachea [4]. It is relatively less common than tracheomalacia (TM) and tracheobronchomalacia (TBM), which are more prevalent in children. The suggested treatment includes inhaled ipratropium and positive pressure ventilation to alleviate symptoms [4]. The effective NIV provides continuous positive airway pressure, effectively acting as a pneumatic stent to limit dynamic airway collapse, alongside intensive respiratory physiotherapy and close monitoring [12].

However, if medical management proves unsuccessful, surgical intervention may be necessary, such as the implantation of an end-bronchial stent [13]. Endobronchial stenting represents a minimally invasive option and may be considered in selected cases with localized airway collapse or as a temporary diagnostic trial to predict response to definitive surgical intervention [12].

Regarding Marfan syndrome, the available evidence is constrained; nevertheless, it is acknowledged that the syndrome can trigger diverse respiratory complications in the lungs [7,8,14]. This includes the weakening of airway walls, which may result in conditions like bronchomalacia and TM. Furthermore, factors such as prolonged intubation and MV, airway trauma during surgical procedures, and infections are also recognized as potential causes of bronchomalacia [14].

The patient's collapse due to respiratory failure was effectively managed. During the intubation process, she showed gradual improvement. In the present case, the diffuse nature of bronchial collapse observed during bronchoscopy, combined with the patient’s recent major cardiac surgery and hemodynamic instability, rendered both endobronchial stenting and surgical airway stabilization inappropriate. The decision for surgery was considered inappropriate due to its high mortality rate, as indicated in Table 1 [15-18].

**Table 1:** Cases of thoracic aortic aneurysm (TAA) and bronchomalacia.

Patient no. (year of publication)	Sex	Age (years)	Genetic disorder	Treatment	Outcome
1 (2010) [15]	Female	59	Unrecognized Marfan’s syndrome	Implantation of an endobronchial stent and advanced second-stage operation	Discharged from hospital
2 (2010) [16]	Female	77	None	Implantation of expandable metallic stents (unsuccessful placement)	Death (pneumonia postoperatively period)
3 (1997) [17]	Female	75	None	Endobronchial stent implantation	Death (eighth day postoperatively, complications)
4 (1996) [18]	Male	76	COPD	Mechanical ventilation and aggressive medical therapy	Death pre-surgery

The medical treatment encompassed intravenous administration of Noradrenaline and Landianol, nebulized bronchodilators, steroids, and antibiotics. From the time of extubation (postoperative day 4), and throughout the period without intubation, the patient's respiratory function was supported with NIV with Continuous Positive Airway Pressure (CPAP). She remained hospitalized in the ICU for seven days, receiving comprehensive care from both doctors and nurses. This holistic approach involved continuous monitoring of her respiratory status, oxygen therapy, and nebulized bronchodilators. Additionally, airway clearance techniques, proper patient positioning, pain management, emotional support, as well as patient and family advocacy and education were diligently provided, and proved lifesaving interventions [19].

## Conclusions

Our case demonstrated that bronchomalacia can lead to total lung collapse in patients with suspected tissue disorders after cardiac surgery. The causes and treatments of this rare condition vary, underscoring the importance of having a working knowledge of available options. The optimal management is conservative NIV, respiratory physiotherapy, and close clinical monitoring. Surgical airway stabilization is generally contraindicated due to the diffuse nature of airway weakness and the elevated risk of complications in connective-tissue disorders. Healthcare professionals, including physicians and nurses in ICUs, should be acquainted with managing this rare respiratory condition following cardiac surgery. Furthermore, a comprehensive understanding of managing young patients with thoracic aortic disease and patients with uncommon genetic disorders is crucial.
